# Whatever Next and Close to My Self—The Transparent Senses and the “Second Skin”: Implications for the Case of Depersonalization

**DOI:** 10.3389/fpsyg.2021.613587

**Published:** 2021-05-31

**Authors:** Anna Ciaunica, Andreas Roepstorff, Aikaterini Katerina Fotopoulou, Bruna Petreca

**Affiliations:** ^1^Institute of Philosophy, University of Porto, Porto, Portugal; ^2^Faculty of Brain Sciences, Institute of Cognitive Neuroscience, University College London, London, United Kingdom; ^3^Interacting Minds Centre, Aarhus University, Aarhus, Denmark; ^4^Research Department of Clinical, Educational and Health Psychology, University College London, London, United Kingdom; ^5^Royal College of Art, London, United Kingdom

**Keywords:** self-awareness, touch, altered states of consciousness, depersonalization, body schema, body image, predictive processing

## Abstract

In his paper “Whatever next? Predictive brains, situated agents, and the future of cognitive science,” Andy Clark seminally proposed that the brain's job is to predict whatever information is coming “next” on the basis of prior inputs and experiences. Perception fundamentally subserves survival and self-preservation in biological agents, such as humans. Survival however crucially depends on rapid and accurate information processing of what is happening in the here and now. Hence, the term “next” in Clark's seminal formulation must include not only the temporal dimension (i.e., what is perceived *now*) but also the spatial dimension (i.e., what is perceived *here* or next-to-my-body). In this paper, we propose to focus on perceptual experiences that happen “next,” i.e., close-to-my-body. This is because perceptual processing of proximal sensory inputs has a key impact on the organism's survival. Specifically, we focus on tactile experiences mediated by the skin and what we will call the “extended skin” or “second skin,” that is, immediate objects/materials that envelop closely to our skin, namely, clothes. We propose that the skin and tactile experiences are not a mere border separating the self and world. Rather, they simultaneously and inherently distinguish *and* connect the bodily self to its environment. Hence, these proximal and pervasive tactile experiences can be viewed as a “transparent bridge” intrinsically relating and facilitating exchanges between the self and the physical and social world. We conclude with potential implications of this observation for the case of Depersonalization Disorder, a condition that makes people feel estranged and detached from their self, body, and the world.

## Introduction

In daily life, we experience ourselves as constantly immersed in an ongoing flow of sensory signals (smells, sounds, images, etc.) emerging from both inside and outside our bodies. These sensations and perceptions scaffold both (a) a *sense of self*, i.e., the subjective first-personal “I” or “self,” bound to my body and distinct from the world and others (Gallagher, 2000; see Qin et al., 2020 for a recent review). And, (b) a *sense of presence*, i.e., the feeling that I am immersed and in direct touch with a real world *here and now* (Seth et al., 2011). Perceptual experiences are traditionally regarded as the fundamental point of contact of an experiencing subject with external, mind-independent^1^ world situated “out there.”

The past decades have witnessed an “embodied turn” in examining perception from a situated, embodied and dynamic viewpoint. The body is not viewed anymore as a mere “material” or “physical” support system for transporting and fueling the mind and brain. Rather minds and brains are designed to support the maintenance and survival of a body within a wider and potentially threatening physical and social environment (Varela et al., 1991; Gallagher, 2005; De Jaegher and Di Paolo, 2007; Thompson, 2007).

In line with this approach, recent work within the influential Predictive Processing (PP) framework suggested that biological agents, such as humans, are “pro-active survival-enabled prediction machines” (Clark, 2013, p. 1) that must optimally maintain their bodily states within the required limits for survival and reproduction purposes^2^ (Friston, 2008; Hohwy, 2013; Fotopoulou, 2015; Seth and Tsakiris, 2018). To complete this difficult task, the human brain generates its own internal self- and world-models by extracting statistical patterns of relevant information (Conant and Ross Ashby, 1970; Friston, 2005; Friston and Stephan, 2007). These self- and world-models are constructed moment-by-moment at various levels of the hierarchical processing (Clark, 2013; Hohwy, 2013).

In this view, the main function of perceptual experiences is ultimately geared toward self-preservation. Perceptions are underpinned at the neural level by dynamically shifting generative models “predicting” what is causing incoming sensory events, based on Bayesian probabilistic “guesses” about the likely causes of any incoming sensory information (Friston, 2008; Hohwy, 2013; Fotopoulou, 2015). Incoming sensory inputs are then contrasted or “matched” against learned (or innate) patterns constituting what are called “predictions.” When a prediction does not match ongoing sensory input, then a “prediction error” results, which may have the effect of updating the prediction.

It has been proposed that a system's ability to attenuate or to “forget” boring (i.e., predictable, unsurprising) information enables the agent to select “newsworthy” information. For example, when picking a ripe cherry from a tree (Limanowski and Friston, 2020), we seem to be very sensitive to the feel of the cherry, as we touch and grasp it. Yet, we are almost insensitive to the feelings of our arm and eye movements while reaching the cherry. However, these inputs are key in ensuring we successfully pick the ripe cherry, and not the green one next to it.

Importantly, a self-organizing system, such as the human body, is most intimately acquainted with *self*-related signals. This means that the problem the brain has to solve is often “not which sensory evidence to *emphasise*, but which to *attenuate*” (Parr et al., 2018; Limanowski and Friston, 2020, p. 8, original italics). One key observation here is that perceptions and beliefs about the self are unique, in the sense that they are “necessarily transparent” (Limanowski and Friston, 2018, p. 5; Ciaunica et al., 2020). Indeed, it has been suggested that in daily life and under normal circumstances, there is a basic, embodied and pre-reflective sense of self that is “transparent.”

As we will see shortly in more detail, the property of “transparency” has been spelled out in a variety of different ways by different theorists (Moore, 1903; Harman, 1990; Tye, 1999; Metzinger, 2003; Sass and Parnas, 2003; Fuchs, 2005; Ciaunica et al., 2020). The basic idea is that transparent processing gives us the feeling of having direct access to what our experiences are about, or directed at. Crucially, this model applies not only to our perception of the external world but also to self-models: “just as a transparent world-model grants the experience of being in immediate touch with the world, a transparent phenomenal self-model… affords the experience of being in immediate relation to a self” (Metzinger, 2003; Limanowski and Friston, 2018, p. 2).

A detailed overview of the PP literature, its advocates and opponents, lies beyond our scope here. In this paper, we retain and build upon the key and largely non-controversial idea that perceptual processing in the here and now is fundamentally geared toward self-preservation (Clark, 2013).

If this is so, then one may pay careful attention to perceptual processing taking place not only in the next second, but literally next-to-my-body. In this paper, we propose to shift the focus on the perceptual processing of proximal sensory inputs (literally “next-to-the-self”), and more specifically on touch which has a key impact on the organism's survival. In line with seminal paper “Whatever next? Predictive brains, situated agents, and the future of cognitive science” by Clark (2013), we suggest that the term “next” should be understood not only at the temporal scale (i.e., what is perceived in the upcoming second) but also from a spatial dimension (i.e., what is perceived literally next to or close-to-my-body).

There are at least two main reasons this shift in focus toward what is happening in proximity to the body. (1) First, as de Vignemont (2018) rightfully notices, the perception of a snake next-to-my foot is essentially different from the perception of the moon in the sky in terms of survival outcome. Indeed, standard accounts tacitly privileged a visuospatial model of perception, which is easily understandable insofar as healthy adults seem to be presented with a continuous visual field devoid of any phenomenological boundary between what is close and what is far (de Vignemont, 2018). Yet, there is growing interest in highlighting the inherent multisensory and proximal nature of perceptual experiences as they unfold throughout the lifespan (Noë, 2004; Ciaunica and Fotopoulou, 2017; Faivre et al., 2017; Fotopoulou and Tsakiris, 2017; Seth and Tsakiris, 2018; Ciaunica and Crucianelli, 2019; Barwitch, 2020).

(2) Second, if our current perceptual experiences are indeed infused and structured by previous “priors” or “expectations,” then it becomes crucial to understand how perceptual experiences unfold, dynamically, from a bottom-up developmental perspective. Indeed, in real life, human beings are not emerging as fully-fledged adults–like Athena famously emerged from Zeus' head–but they gradually develop from cells to a human body within another human body (Fotopoulou and Tsakiris, 2017; Ciaunica, 2019; Ciaunica and Crucianelli, 2019; Quintero and de Jaegher, 2020; Ciaunica et al., 2021). As we will see shortly, endorsing a dynamic bottom-up viewpoint points us to the idea that touch, before vision, plays a fundamental role in constituting self- and world-models in early life and beyond (Fotopoulou and Tsakiris, 2017; Ciaunica and Crucianelli, 2019).

Indeed, humans have proximal and “direct sensible acquaintance” (James, 1890) with others' bodies (via skin-to-skin interactions) well before they meet others' minds (via face-to-face and eye gaze interactions) (Ciaunica and Fotopoulou, 2017; Ciaunica et al., 2021). We argue that paradoxically, precisely because tactile proximal inputs are ubiquitous, they tend to be “transparently” processed “in the background,” typically unnoticed and taken for granted. Yet, they constitute the fundamental “invisible” roots of our sense of self and sense of presence in the world.

We unpack these ideas below, and we proceed as follows:

In the Perceptual Awareness and the Transparent Experiential Background section, we define the notion of “transparency,” and we explore the phenomenological idea that proximal sensory inputs (e.g., olfactory, proprioceptive, tactile, interoceptive) are blended to form a tacit “background existential orientation” (Ratcliffe, 2008). Specifically, we focus on tactile experiences as pervasive yet essential components of our everyday life, forming a baseline and transparent experiential background.

The Getting Perception off the Ground: The Basic Proximal Senses section motivates the claim that tactile experiences may precede more sophisticated forms of detached and distal perceptual awareness—such as visuospatial perception (e.g., seeing an apple). Indeed, well before humans are able to recognize themselves in a mirror and to perceive themselves from a visuospatial distal perspective, they experience themselves and their surroundings via proximal senses. We review empirical developmental findings pointing to the key role of touch in constituting perceptual experiences in early life and beyond.

In the (Un)Covering our Body—Extending the Transparency to the “Second Skin” section, we tackle a relatively overlooked aspect of our tactile experiences, namely, the fact that with rare exceptions, humans in modern societies spend most of their lives having their bodies closely enveloped by clothes. We focus on tactile experiences that extend to the immediate objects/materials that envelop closely our skin. Following the seminal “extended mind” thesis (Clark and Chalmers, 1998; Kirchhoff and Kiverstein, 2019), we argue that these materials may be conceived as a “second skin” or “extended skin” that underwrite what we will call here “extended body-image” and “body-schema” (cf. Gallagher, 2005).

Finally, in The Transparent Bridge—Bodily Connectedness Through Proximal Senses section, we suggest that tactile experiences–mediated by the skin and the “extended skin” –may be viewed as a “transparent bridge” intrinsically relating and facilitating exchanges between the self and the physical and social world. We build upon the observation that the skin and tactile experiences have an inherent dual function: it simultaneously separates and relates our bodily self to the physical and social world. Touch mediates indeed our self-presence in the world. We thus hypothesize that close tactile engagements with the social and physical environment play a fundamental role in shaping both our sense of self and sense of presence or immersiveness in the world. We briefly discuss some potential implications of this observation for the case of Depersonalization Disorder, a condition that makes people feel estranged and detached from their self, body, and the world. We conclude with some potential implications of our hypotheses for designing bodily and interactive interventions aiming to alleviate the symptoms of depersonalization. If our hypotheses are correct, then it is paradoxically by inviting depersonalization people to “forget” their self and to get closer to the world and others that they may start to get closer to their “lost” self.

## Perceptual Awareness and the Transparent Experiential Background

Perceptual awareness is typically described as having a polarized subject–object structure whereby an experiencing subject perceives an “external” world/object “out there.” Traditionally, it has been proposed that one becomes aware of one's self when one is able to perceive oneself as an object of one's awareness and to see oneself through others' eyes (Carruthers, 1996). For example, when I see myself in a mirror, I recognize myself as an individual distinct from the world and others. But also, I can see myself as others see me, from a third-person perspective.

However, as James (1890) pointed out, before we see ourselves as others see us, we perceive ourselves and our bodies through feelings. The most fundamental way to get to know oneself is through feelings, not thought: “For this central part of the Self is *felt*… It is at any rate no *mere ens rationis*, cognized only in an intellectual way, and no mere summation of memories or mere sound of a word in our ears. It is something with which we also have *direct sensible acquaintance*… when it is found, it is *felt*; just as the body is felt” (James, 1890, p. 298–299 original italics; bold our emphasis).

Indeed, a long-standing phenomenological tradition pointed out that among the objects that an experiencing subject perceives in the world throughout her life, the body has a special status (Merleau-Ponty, 1962). For example, Husserl famously wrote that the body is not just an object that is perceived but also that through which we perceive: “The Body [*Leib*] is, in the first place, the *medium of all perception*; it is the *organ of perception* and is *necessarily* involved in all perception” (Husserl, 1989, p. 61). The body follows the experiential subject everywhere, like a shadow, for better and for worse (Legrand, 2006).

In line with these ideas, it has been proposed that all experiences include a “background existential orientation” constituted by bodily feelings: “the feeling *is* the way in which one finds oneself in the world and the way in which one finds oneself in the world participates in all experience, albeit as something that is usually pre-reflectively taken for granted” (Ratcliffe, 2008, p. 140).

The idea of an existential bodily background has been highlighted in neuroscience research. For example, Damasio writes:

“I am postulating another variety of feeling which I suspect preceded the others in evolution. I call it *background feeling* because it originates in ‘background’ body states rather than in emotional states. It is not the Verdi of grand emotion, nor the Stravinsky of intellectualized emotion but rather a minimalist in tone and beat, the feeling of life itself, the sense of being. (…) The background feeling is our image of the body landscape when it is not shaken by emotion. (…) I submit that without them the very core of your representation of self would be broken” (Damasio, 1994, p. 150–151, our italics).

The lived body is the feeling body that remains tacitly, and one could say “transparently” in the background^3^. In the remainder of this paper, we build upon the idea that these bodily feelings constitute a pervasive and “transparent” experiential background, which can be attended or altered, i.e., “opaque” or “broken” (Fuchs, 2005; Ciaunica et al., 2020).

Transparency can be intuitively grasped via the “window” metaphor: a clear and transparent window glass or sliding door can give us the illusion of an unmediated access to the outside world. However, although we subjectively feel that we are directly in touch with one's inner self and the outer world, in reality, our experiences are *mediated* through certain states or processes that are transparent, pervasive, and tacitly taken for granted (Fuchs, 2005; Ciaunica et al., 2020).

As we saw earlier in the Introduction section, the notion of “transparency” has been theoretically spelled out in different ways by different theorists (Moore, 1903; Harman, 1990; Tye, 1999; Metzinger, 2003), and a detailed review of these accounts lies beyond the scope of this paper. Here, we restrict our focus on the property of transparency of perceptual experiences. To get a clear intuitive grip on this idea, consider the following example: I am on a street, and I hesitate to turn my head left or right. Eventually, I turn my head left, and I realize that I chose this direction, because I have perceived with the corner of my eye the label of a French bistro. Suddenly, I realize that I am hungry, and the reason why I turned my head into that direction is because my brain, concerned by my bodily survival, detected some relevant “transparent” background information, that is now brought forward, at the surface of my awareness (the smell of delicious French cuisine, say). Consequently, I engage in self-regulatory behavior, and I walk toward the bistro and order escargots.

Here, we restrict our focus on the property of transparency of experiences (and leave aside the property of transparency of mental representations^4^ or processes). Note that it is important to distinguish, in our view, between (a) what is non-conscious but can be in principle consciously attended (brought into the focus of the attention; e.g., the visual stimuli of a bistro label or the interoceptive signals of my hungriness); and (b) what is non-conscious and cannot be consciously attended (e.g., the firing of my neurons while I perceive the French bistro; or the metabolic exchanges between my blood and my organs). Only the former information, but not the latter, can be processed “transparently” because only (a) but not (b) can be attended, and thereby become “opaque.”

In what follows, we explore the idea that our tacit “background existential orientation” is essentially underpinned by proximal sensory inputs (e.g., olfactory, proprioceptive, tactile, and interoceptive) that are blended to form an experiential “transparent” background. These signals are so pervasive and yet so essential to our being in the world that they seem to form a baseline and taken for granted experience. Specifically, we focus on touch^5^ and tactile experiences for three interrelated main reasons. First, touch is mediated by the skin, the oldest and widest organ in terms of dimensions and functions (Montagu, 1971; Field, 2001; see Gallace and Spence, 2010 for a review). By providing the organism with the most primitive means to “meet” and perceive the world, tactile experiences may constitute thereby the most ubiquitous and basic experiential background.

Second, the skin mediates the boundary between the self and the outer world, and as such, tactile experiences display an inescapably dual “touchant/touché” structure (Merleau-Ponty, 1962). By gaining information about the world via touch, the subject inherently gains information about her “self” too. Indeed, while proprioception, kinaesthesia, and interoceptive of visceral inputs are phenomenologically inextricable components of the existential background feeling, tactile experiences because they play a special relational or dual role: “tactual perception” (Ratcliffe, 2013) is closely associated with or partly constituted by perception of one's body: one cannot perceive the world tactually without perceiving oneself in the process. As Merleau-Ponty (1962, p. 316) famously pointed out, while vision “presents us with a spectacle spread out before us at a distance,” in perceiving the world through touch, “I cannot forget in this case that it is through my body that I go to the world” (1962, p. 316). By directly mediating the boundary between body and world, the skin inescapably distinguishes yet relates body and world, as the two faces of the same coin. As Martin notes (Martin, 1992, 1993, 1995), tactile feelings can simultaneously be perceptions of body and/or of world.

Lastly, but importantly, touch plays a key exploratory and social bonding role, which confers on it a sense of “closeness”: we touch things to make sure they are real, and we touch people to make direct and close contact with them. As Fulkerson (2014) notes, what distinguishes tactual experience from the other senses is the fact that the former involves “exploratory binding”; it relies upon actively manipulating (and being manipulated by) the environment.

Up to now, we have motivated the claim that the body–world relationship mediated via tactile perception remains tacitly and ubiquitously in the background and the sense of self may be inseparable from this relationship. In the next section, we suggest that close tactile experiences may precede developmentally more sophisticated forms of detached and distal perceptual awareness—such as visuospatial perception (e.g., seeing an apple). Well before humans are able to recognize themselves in a mirror and to perceive themselves from a visuospatial distal perspective, they experience themselves via proximal senses, such as tactile experiences.

## Getting Perception Off the Ground: the Basic Proximal Senses

There is a growing consensus in philosophy, psychology, and cognitive neuroscience that multisensory information about the body plays a central role in structuring our basic sense of self (Gallagher, 2000; Blanke and Metzinger, 2009). Specifically, the interplay and coupling between (a) exteroceptive (e.g., vision, audition) and (b) interoceptive senses (e.g., temperature, pain, cardiac signals, breath, etc.) is a key component of our sense of self (Park and Blanke, 2019). More recently, the PP framework proponents argued that the basic experience of being a self is the result of an ongoing inferential process based on a generative model centered onto the bodily self (Apps and Tsakiris, 2013; Hohwy, 2013, 2020; Limanowski and Blankenburg, 2013; Seth, 2013; Limanowski and Friston, 2018, 2020).

Biological agents, such as humans, act as “self-evidencing” systems aiming at maximizing evidence for their self-model as they minimize prediction errors (Hohwy, 2014). In short, self-organizing systems need to constantly “check” and “prove” themselves that they exist in a dynamic, constantly changing and noisy world. The self is an inferred model of endogenous, deeply hidden causes of behavior. It has been proposed that self-modeling can be described as a two-step process: (i) first finessing a model of the self and then (ii) engaging that model in action, which spirals into further finessing of the model, and further action, and so forth (Hohwy and Michael, 2017).

However, one basic yet overlooked aspect of current embodied and PP approaches in both philosophy and cognitive neuroscience is that brains (and minds), and human bodies, first develop *within* another human body. The most basic models of perceptions and actions emerge already *in utero* (Ciaunica, 2019; Ciaunica and Crucianelli, 2019; Quintero and de Jaegher, 2020; Ciaunica et al., 2021). Crucially, while not all humans will have the experience of being pregnant or carrying a baby, the experience of *being carried and growing within another person's body* is universal^6^ (Ciaunica et al., 2021).

In the remainder of this section, we provide evidence illustrating that it is touch (and not vision) that is one of the first of our senses to develop and affords us thereby with our most basic and earliest means of “meeting” and perceiving both the self and the external world (Ciaunica and Fotopoulou, 2017; Fotopoulou and Tsakiris, 2017; Ciaunica and Crucianelli, 2019).

In the womb, fetuses spend a significant amount of time in tactile exploration of the boundary between innervated and non-innervated regions (Mori and Kuniyoshi, 2010; Piontelli, 2014; Hata, 2016). For example, fetuses frequently touch certain body areas, such as the lips, cheeks, ears, and parietal bone, creating a self-stimulatory pattern, which enhances innervation. Importantly, when the fetus touches the forehead, innervation increases, and the boundary migrates (Piontelli, 2014). This allows the fetus to move on in touching a new innervated boundary, and the cycle repeats until the whole body is fully innervated (Piontelli, 2010; Delafield-Butt and Gangopadhyay, 2013). Additionally, when the fetus touches itself, the placenta, or a co-twin, it develops different kinematic and tactile patterns that emerge, which differ in pressure, acceleration, and directedness (Hata, 2016).

For example, Castiello et al. (2010) investigated the kinematic profiles of movements in five pairs of twin fetuses by using four-dimensional ultrasonography during two separate recording sessions carried out at the 14th and 18th weeks of gestation. They showed that by the 14th week of gestation, twin fetuses do display not only movements directed toward the uterine wall and self-directed movements but also movements specifically aimed at the co-twin, the proportion of which increases between the 14th and 18th gestational weeks. They also noted similar kinematic profiles for movements directed toward the co-twin and self-directed movements aimed at the eye-region, i.e., the most delicate region of the body. They concluded that performance of movements toward the co-twin is not accidental: already starting from the 14th week of gestation, twin fetuses execute movements specifically aimed at the co-twin. This has been reported in singleton pregnancy as well, where kinematic studies seem to suggest that motor planning is in place by 22 weeks of gestational age (Zoia et al., 2007).

Moreover, it has been shown that maternal touch of her own abdomen increases arm, head, and mouthing movements in the fetus (Marx and Nagy, 2015), and that maternal touch has more impact than maternal voice in the fetus' movements. Notably, tactile interactions require the “toucher” and “touched” to be physically proximal, to “share” the experience of touch (passive or active), and it is often accompanied by a cascade of other sensorial information, such as the smell of the other person, the sound of the tactile contact on the skin (think of the “noise” made by a kiss), and temperature of the other body. Given the richness of information provided by tactile interactions, it has been hypothesized (Ciaunica and Fotopoulou, 2017; Fotopoulou and Tsakiris, 2017) that social touch might represent a fundamental step in the development of both self- and other-awareness, as well as self-other distinction (see McGlone et al., 2014 for a review).

Specifically, one type of pleasant touch, the so-called affective, or sensual touch (i.e., slow, caress-like touch mediated, among other, more classic tactile fibers, by the C Tactile afferent system, Löken et al., 2009; Kirsch et al., 2020), has been re-defined as an interoceptive^7^ modality since its primarily functional role seems to be to provide information about the homeostatic and emotional effects of touch, rather than the properties of what or who is being touched. It has also been shown that the activation of these CT receptors on the skin may specifically relate to the positive consequences of interpersonal touch (see McGlone et al., 2007), such as reducing feelings of social exclusion (von Mohr et al., 2017), soothing pain (Krahé et al., 2016; von Mohr et al., 2018), and communicating social support (Kirsch et al., 2018). Indeed, related findings suggest that this system may be specialized for processing not only affective touch but also specifically *social affective touch* from early on in life (see Morrison et al., 2010 for a review). Specifically, the fetus is entirely covered in fine hair (i.e., lanugo hairs), and it has been suggested that fetal movement in the amniotic fluid might directly stimulate CTs afferent, which are known to activate the hypothalamus and insular cortex, promoting an anti-stress effect via realize of oxytocin and stimulating the fetal growth (Bystrova, 2009).

A second key example of early sensorimotor coordination is self-touch and tactile perceptions. While a significant body of research focused on the effect of auditory inputs (such as maternal voice or music) on the fetal development, it is important to bear in mind that in the womb, the most developed sensory systems in fetuses are the tactile and olfactory ones, not the visual or auditory ones. This suggests that the most basic way to perceive oneself and the world is mediated through proximal modalities (Ciaunica and Fotopoulou, 2017), such as touch, interoception, and olfaction (Ciaunica and Crucianelli, 2019).

After birth, infants receive constant and proximal tactile stimulations, which significantly reduce infant's stress and increase positive affect (Stack and Muir, 1992; Bellieni et al., 2007). Affective touch is believed to play an important role in the creation and maintenance of social bonding^8^ (see Morrison et al., 2010 for a review) and more recently to the sense of body ownership (Lloyd et al., 2013; van Stralen et al., 2014) and self-identity (Panagiotopoulou et al., 2017).

We can summarize the argumentative backbone developed so far as follows: if we grant the premise that the perception of incoming “whatever next” (Clark, 2013) sensory information emerges from the processing of “whatever before,” i.e., priors or “expectations,” then one needs to step back and have a closer look at how perception gets off the ground from the outset. If we endorse a bottom-up developmental perspective, then empirical work points us to the idea that (discriminative and affective) tactile inputs play a fundamental role in constituting perceptual experiences in early life and beyond.

In the next section, we turn our attention to the special relationship between touch, body, and what is next-to-our-skin. We introduce the key notion of “the second skin” or “extended skin” and explore them in relation to materials situated close to our skin, i.e., our clothes. We claim that they constitute what one may call, following Gallagher (2005), “extended” body-image and body-schema. We turn to this discussion now.

## (Un)Covering Our Body—Extending the Transparency to the “Second Skin”

Most of the time, in our daily lives, our body is covered with clothes. Textile are pervasive material objects enveloping closely our skin. Clothes may thus play a peculiar role in our bodily experiences. First, they seem to be at the same time visible and invisible. Visible because they are tangible and on display on our bodies and the bodies of others. Invisible, because we tend to “forget” them in the background and to process them “transparently” as defined above. Second, materials that envelop our body allow individuals to closely relate to and exchange with the external physical and social world.

Throughout human history, individuals ingeniously made use of various materials close to their bodies—for protection, comfort, and aesthetic aspects. Particularly, textiles and clothes, which throughout the “long and intimate” (O'Connor, 2010) connection with people, were key in symbolic representations of self (e.g., identity, gender, age, class, political views, as well as a variety of social values; Guy et al., 2001; Weber and Mitchell, 2004; Mida and Kim, 2015), in the reproduction of social order (e.g., in making social differences visible, see Entwistle, 2000; Breward, 2003; Crane, 2012), in embodying culture (memory, history, and identity), and in “transforming, protecting, and healing the human body” (O'Connor, 2010; see also Hansen, 2004; Brumfield, 2006). Many times, textiles have been used with varying degrees of explicitness as a medium to record history or to “provide a focus for the expression of conflict or reflect commentary on current affairs” (Henderson, 1990). The latter can be noticed, for example, in more codified manners, such as in Ganseys^9^, or in a very explicit manner in t-shirts portraying rock music bands or political messages, a style very much influenced by Vivienne Westwood's collaboration with Sex Pistols in the 1970s. Hence, we can view clothing as “the furniture of the mind made visible” (Laver in Barnard, 2007, p. 2).

This long and intimate relationship also endowed textiles with magical, and at times humanlike, characteristics in people's imaginaries. Textiles may be perceived as being “alive” (e.g., designers describe textiles as an animated, living thing with whom they engage in conversation to define what a textile wants to be as a garment; for a full account, see Petreca et al., 2015), or, as observed by Henderson (1990, p. 1,588) may be seen to “have supernatural properties” (e.g., the invisibility cape in Harry Potter novels), or “may be used as a metaphor for life” (e.g., “Penelope rips out her weaving nightly in order to arrest the flow of time”), or even to be “imbued with the characteristics of their wearers or impart special qualities to them” (e.g., the widespread culture of celebrities' clothes auctioning, where pieces are sold at exorbitant prices^10^).

The clothes lie at the interface of the body and its social presentation, and they “signify to the wider world who and what a person is trying to express” (Salgado-Montejo et al., 2018). This symbolic level is rooted on the physical touch basis of the experience of wearing, and we saw earlier that this long and intimate, proximal experience grants textiles “magical” perceptions of “being alive” and “being imbued with the characteristics of their wearers.” Indeed, clothes have a unique capacity to provide immediate feedback to an individual, the wearer, affirming their sense of identity at a directly embodied level (Twigg and Buse, 2013). Clothes can express largely unconscious aspects both of an individual and of a group psyche (Salgado-Montejo et al., 2018) and form a part of our non-verbal communication, or what is defined by Lurie (2000), as a “universal tongue.”

What we wear reflects our personality, or undressed self, and therefore, it is a material manifestation of our beliefs and behaviors, or “how we think and how we live” (Salgado-Montejo et al., 2018, p. 2): “enclothed cognition is a bidirectional phenomenon, with the wearer selecting clothes that reflect his/her personality and clothing having an influence on cognitive processing and behavior” (Salgado-Montejo et al., 2018, p. 2). For example, it has been suggested that people are efficient and effective in forming impressions of someone's personality via clothing, specifically shoes (Gillath et al., 2012). Furthermore, clothing can influence self-perception. People wearing formal clothing considered themselves as competent and rational (Hannover and Kühnen, 2002; Peluchette and Karl, 2007) and also have enhanced abstract cognitive processing (i.e., comprehensive mental representations) and increased perceived social difference and power (Slepian et al., 2015).

In what follows, we suggest that the clothes may be described as a “second skin” and as such they are an essential component of the transparent experiential background defined in the Perceptual Awareness and the Transparent Experiential Background section. They “extend” the body's schema and image. In his seminal formulation, Gallagher defined (i) “body image” as “a complex set of intentional states and dispositions, perceptions, beliefs, and attitudes, in which the intentional object is one's own body” (Gallagher, 2005, p. 25). By contrast, (ii) the “body schema” is a “non-conscious performance of the body, i.e., a performance that is not an intentional object present to my consciousness” (Gallagher, 2005, p. 548).

Interestingly, he points out that the body-schema can include tools and artifacts as well:

“the carpenter's hammer becomes an operative extension of the carpenter's hand, or as noted, the body schema extends to the feather in the woman's hat (see Gorman, 1969, p. 15). The body schema is an active, operative performance of the body, rather than a copy, image, global model, or conception of the existing parts of the body. The schema is the body as it actively integrated its position and responses in the environment. (…) The body schema, understood this way, is not the perception of ‘my’ body; it is not the image, the representation, or even the marginal consciousness of the body. Rather is it precisely the style that organises the body as it functions in communion with its environment” (Gallagher, 1986, p. 548–549).

The extended mind thesis famously defended by Clark and Chalmers (1998) claims that the cognitive processes can be “offloaded” or “extended” to reach beyond the boundaries of individual, such as to include as proper parts aspects of the individual's physical and sociocultural environment (see Kirchhoff and Kiverstein, 2019 for a recent review). Kirchhoff and Kiverstein (2019) use the example of a spider and its web: the latter becomes transparent for the spider and perceives its prey through the web. But how about bodily sensory processes? Can they be “offloaded” or “extended” to reach beyond the boundaries of the skin?

We suggest that some perceptual bodily experiences can be extended through (i.e., transformed into) components that reach beyond the sensory organ's states (e.g., the skin) into what one may call an “extended skin” or a “second skin.” Hence, the transparency of tactile experiences may be “offloaded” into the materials close to the skin. The notion of “offloading” refers to the use of artifacts (here clothes) to manage and convey information about the self. For example, clothes may be regarded as a key component of what one may call an (i) “extended” body-schema (e.g., regulating body temperature via warm clothes) and (ii) “extended” body-image, contributing to the constitution of one's social (narrative) self via social signaling (e.g., wearing a red T-shirt to signal that we support Arsenal football club; see Colombetti, 2016).

The idea is that clothes are typically processed transparently, in the background. This is because, by being so close to our skin, they “inherit,” so to speak, the pervasiveness and transparency of tactile experiences, which then “extends” to the clothes. The skin being the largest of our sensory organ and given that most of the time, most of our bodies (and hence skin) are covered by clothes, this means that we may tacitly “sense” the world through this interface. We can thus speak of an “extended transparency.”

There is a robust body of work illustrating that our representation of our own body and our bodily sensations does not end on our skin; instead, the proximal space around the body, known as the peripersonal space (PPS), is included in our multisensory representation of the body (see Serino, 2019 for a recent review). Early neurophysiological studies in primates identified a specific population of multi-sensory neurons that selectively responded to external stimuli, but only when the somatosensory, visual, or auditory stimulation occurred close to and not far from the body (Rizzolatti et al., 1981). Subsequent neuropsychological (e.g., di Pellegrino et al., 1997; Làdavas, 2002; di Pellegrino and Làdavas, 2015), psychophysiological (e.g., Maravita et al., 2002, 2003), and neuroimaging (e.g., Bremmer et al., 2002; Blanke et al., 2015; Cléry et al., 2015) studies showed that the processing of tactile information is strongly affected by external stimuli presented close to the body, further cementing the notion that a similar system in humans exists.

This space representation is thought to be dynamic rather than static, so as to maximize the processing of relevant events for the self, expanding, and shrinking depending on environment specificities. Moreover, sociological and anthropological studies have revealed the role of PPS in the type and strength of social and cultural interactions (Hall et al., 1968; Sommer, 1969; Hall, 1990; Felipe and Sommer, 2017). More recently, social neuroscience brought these ideas together to show that interacting proximally with another person influences how we perceive not only our own body but also the space around it, depending on our precise relationship with that person and how much we trust them (Heed et al., 2010; Teneggi et al., 2013).

A detailed discussion of the fascinating topic of the role of PPS in constituting self-representations and our sense of self lies beyond the topic of this paper^11^. Here, we retain the idea of a literally “close relationship” between clothes and bodily self-experiences.

Note that there is an important distinction between “extended body schema” in tool as opposed to clothes (daily) experiences^12^. Take for example a blindman's stick. A successful tool use in this case implies that the individual successfully *attends to* the tip of the stick in order to explore and perceive its environment: she/he can “see” with the stick. The same goes for the carpenter's hammer: he needs to attend to the hammer's contact with the nail in order to hit it correctly. In some cases, the tools may become transparent too^13^: think of a person expertly typing on a laptop keyboard while focusing entirely on the letters appearing on the screen. However, the link between the keyboard and the letter appearance needs to be available for attention, in order to correctly perform the task. If the letters appear on the screen with a delay after I have hit the keyboard, then the transparency breaks down, and I may need to bring my attention to the keyboard itself. In other words, there is an instrumental, functional role in the tool use that requires attention in some way or another.

By contrast, everyday clothes may blend into the background unnoticed. They also need to be comfortable such as we can afford to dis-attend and “forget” about them. In other words, to process them via what one may call an extended transparent experiential background. Unless these proximal tactile experiences become unpleasant or uncomfortable (rough materials, itchy, dry skin), in which case they become “opaque” and emerge at the surface of our awareness. There are cases where clothes are deliberately brought into the foreground, to signal power and social status: think of a king wearing a heavy ermine mantle. The key idea here is that by mediating the boundaries between our bodies and the environment, clothes also seem to have an ambivalent role: they are simultaneously *covering* the intimacy of the self while *uncovering* the self to signal status and power to the public eye.

Up to now, we focused on proximal perceptual experiences as mediated by touch. We took the example of clothes close to our body and suggested that tactile experiences mediated via the skin and the “second skin” become pervasive, transparent and allows what Gallagher called above a “communion with its environment”.

In the last section of our paper, we examine some of the implications of shifting the focus from visuo-spatial to proximal perceptual experiences in relation to our sense of self and sense of presence in the world. Specifically, we briefly describe the case of Depersonalization, a condition that makes people estranged and detached from their self, body, and world. We discuss some empirically testable hypotheses, to be developed in the future work, highlighting the key role of proximal tactile interactions with the environment on one's sense of self and sense of presence in the world. If correct, our hypotheses may fruitfully contribute to designing body-based and dynamic interventions helping to alleviate feelings of self-estrangement and social alienation in people suffering from depersonalization symptoms.

## The Transparent Bridge—Bodily Connectedness Through Proximal Senses

While traditionally theorists have been concerned mainly with the subject–object polarity in describing perceptual experiences, throughout this paper, we aimed at drawing attention at what is happening *in between* these two poles. For this reason, we proposed to shift our focus from visuo-distal perception (easier to structure around a subject/object axis) to proximal perception (and particularly on touch), where the key role of mediating boundaries becomes more evident. Contrary to the standard approach that views the skin (and tactile experiences) as a mere border separating the self and world, here we propose that the skin (and its extended version, “the second skin,” i.e., the clothes) simultaneously and inherently distinguishes *and* connects the bodily self to its environment.

We motivate this shift in light of the new and highly influential PP theories of perception. As we saw in the Perceptual Awareness and the Transparent Experiential Background section, one key idea behind embodied cognition and PP accounts is that perceptual experiences are deeply depended on dynamic engagements and reciprocal interactions with others and the environment (Gibson, 1979; Schilbach et al., 2013). If this approach is correct, then our sense of self and presence or immersiveness in the world may crucially depend on how we relate with our social and physical surroundings. To put it in a slogan: the more we interact with the world and others, the more we gain information about ourselves.

Crucially, human bodies do not emerge in a vacuum. Indeed, one fundamental yet overlooked aspect of our embodiment is that the human organism emerges and develops *within* another human body. While the experience of pregnancy has been traditionally linked to a certain category of individuals (pregnant persons), to date, all humans shared their bodies with the body of another person. Consequently, one needs to adapt and extend the notion of embodiment to reflect the fundamental and universal body-within-a-body case, what it has been termed co-embodiment (Ciaunica et al., 2021).

The regulation of the two agents' states–and particularly the need to maintain physiological stability despite a fluctuating and unpredictable environment–is actively negotiated between the two organisms that share for a given amount of time (typically 9 months) common bodily and environmental resources. Importantly, humans start to perceive themselves and to relate to their close environment, already in the womb, via proximal senses (such as touch) way before vision. After birth, human babies are unable to self-regulate their own homeostatic balance; hence, they fundamentally depend on close physical interactions with dedicated caregivers for survival (Ciaunica and Fotopoulou, 2017; Fotopoulou and Tsakiris, 2017; Ciaunica et al., 2021). As toddlers and adults, we actively explore our proximal surroundings and touch objects to make sure they are real. Since all humans have started their experiential journey as babies within another's body, the co-embodied and relational aspect of perceptual experiences may constitute the basis of our fundamental sense of self.

While there is growing awareness that perceptual experiences are multisensory in nature (Faivre et al., 2017), in this paper, we narrowed our focus on the inescapably dual *touchant/touché* structure of the tactile experiences. This reciprocity and duality place touch in a key position highlighting the inherent relatedness of our bodies with its close physical and social world. We propose to label this overlooked relational background mediated by the proximal senses–and particularly by touch–as a “transparent experiential bridge” that is there without us being aware that it is there. One key question that further work needs to address is the nature of the link between the phenomenological sense of self and this experiential bridge. How does this relationality of our experiences at their most primitive stages fits within the traditional picture of pre-reflective self-awareness or minimal self as being fundamental to all experiences?

While we cannot do justice to this question in this paper, the remainder of this section, we would like to suggest that in daily life, healthy humans constantly use this transparent bridge to communicate and to relate with the world and others, without paying too much attention to its “invisible” and “transparent” underlying structure and pillars, so to speak. However, the importance of this key relatedness becomes apparent especially when this “transparency” breaks down and people start feeling unreal and disconnected from their selves, bodies, and the world^14^.

As Ratcliffe rightly points out, people usually talk about a self as distinct from the body whenever they feel something is disrupted or amiss with the self: something that has gone wrong or something that is lacking. To use our initial metaphor: we notice more easily the existence of a transparent window when its glass cracks (Ciaunica et al., 2020). When the mediating function of transparent senses gets disrupted, people may feel disconnected from their self, body, and world (Ciaunica et al., 2021).

Initially described by Dugas in 1898 (Sierra and Berrios, 1998), Depersonalization/Derealisation Disorder (DP/DR) is a condition characterized by profound alterations of one's sense of self (Sierra and David, 2011), typically inducing distressing feelings of detachment or estrangement from one's self (depersonalization) and/or one's surroundings (derealization) (DSM IV-TR fourth edition, text revision 2000)^15^. DP/DR typically co-occurs in association with highly traumatic events or as symptoms of anxiety, panic, and depression.

These dramatic alterations are typically experienced as a “split” or a “fracture” between an external observing agent and an observed self, body, and world: “My perception felt as though it had been drawn back inside my head, almost as though I was looking at the world from the back of my head, and could see the back of my own eye sockets. (…) Essentially, it felt like there was a divorce or fracture between the world and me so that although my body was still in the world, my mind was only an observer” (Ciaunica et al., 2020, p. 6).

The experienced self-split or self-detachment occurs on multiple levels, as it is associated with (a) detachment from one's body or body parts (low-level sensory and bodily aspects of the self); (b) detachment from one's subjective feelings and emotions (experiential aspects); and (c) disconnection from one's personal stories, memories, thoughts, and future plans, often described by sufferers as a lack of a narrative^16^ or a “plot” in one's life (Ciaunica and Charlton, 2018). The overall impact of this “self-split” makes people feel “not fully real” (Simeon and Abugel, 2008; Sierra, 2009; Medford, 2012).

The prevalence of DPD is around 1–2% in the general population (Hunter et al., 2004), with onset typically occurring before age 25. Strikingly, feelings of depersonalization are the third most common psychological symptom reported in the general population (after anxiety and low mood), especially among young people (Simeon et al., 2003). Yet, the underlying neural and computational mechanisms as well as its phenomenological markers remain poorly understood (see Seth et al., 2011 for an early attempt).

The experience of a “split” between the self and the body–strikingly described as feeling trapped in one's head (mind) and outside one's body (Ciaunica et al., 2020)–is one of the most frequently cited symptoms in DPD (Sierra and David, 2011). Sierra (2009) lists four prominent types of anomalous body experiences in DPD: (1) lack of body ownership, (2) feelings of loss of agency, (3) disembodiment feelings, and (4) somatosensory distortions. Empirical evidence for this disrupted bodily sensory processing comes from studies that report disrupted physiological responses in patients with DPD, compared with healthy participants (Sierra et al., 2002; Owens et al., 2015; Dewe et al., 2018). DPD has also been linked to disrupted activity in neuronal regions underlying somatic processing (Medford et al., 2012; Lemche et al., 2013) and the vestibular system (Jáuregui Renaud, 2015), which is responsible for providing information about the body's position in space (Ferrè and Haggard, 2016).

A detailed discussion on this condition would lead to a substantial digression (see Simeon and Abugel, 2008; Sierra, 2009; Sierra and David, 2011; Medford, 2012; Billon, 2016; Ciaunica et al., 2020). Here, we propose to narrow our focus on relational aspects between the self and the world, dramatically reported by a patient as follows:

“When the depersonalization is very deep, I still seek to ‘be’ someone else because it feels like that constant source of interaction is the only thing that allows me to maintain a connection with the world. I'll also seek physical contact with whoever I'm with. It almost feels as though I need to be that other person because my own sense of self is not strong enough in that moment to sustain me” (Ciaunica and Fotopoulou, 2017).

This observation may be crucial for potential interventions and therapies designed to make DPD people aware of what *still connects* them with their self, body, and the world, despite the fact that they feel dramatically alienated. Specifically, future work needs to disentangle and contrast the role of proximal vs. distal senses in shaping the experience of being a self-present in the world and fully connected with one's body.

If our argumentation throughout our paper is correct, then future research needs to assess whether active engagements with the world and others via proximal (tactile and multisensory) interactions enhance the sense of self, realness, and presence in people with DPD. By contrast, we speculate that distant visuospatial experiences–such as seeing/recognizing oneself in the mirror–will enhance feelings of estrangement and self-detachment, of being inside one's head. Moreover, we hypothesize that close and dynamic physical and synchronous interactions with their environment will make DPD people feel more present in their bodies, and less “trapped” in their minds. This is because, paradoxically, in order to get closer to one's self, one needs to be able to “forget” oneself (Ciaunica, 2020, submitted) and to be able to open to the world and others, via proximal tactile interactions.

The accent thus needs to be put on what connects us to ourselves and the reality, as opposed to what separates us from it, especially when we feel isolated and alienated. To use our metaphor, even if the transparent bridge that connects people with their self, body, and world is shattered, it is important to stress that it remains available and *can* be crossed. As Ratcliffe insightfully notes: “talk of feeling detached from body and world might best express an all-pervasive feeling of estrangement but, importantly, that feeling is *itself* a way of experiencing the body–world relationship and so one has not actually escaped from body and world at all” (2008, p. 131). We must thus use this fundamental connectedness to the world as a powerful tool to repair the “lost” connectedness to one's self.

## Conclusion and Future Directions

While traditionally perceptual experiences have been described as having a subject–object polarity, throughout this paper, we aimed at drawing attention at its relational aspect, i.e., what is happening *in between* these two poles, especially when the sensory signals are proximal as opposed to distal. Shifting focus from visuo-distal perception (easier to structure around a subject/object axis) to proximal perception (and particularly on touch) allowed us to outline the key relational role of tactile interactions with the physical and social environment. We motivated this shift in light of the new and highly influential PP theories of perception. If indeed whatever we perceive now is infused by whatever is perceived before, then it becomes crucial to endorse a dynamic and situated viewpoint in examining perception.

We argued that the term “next” here must include not only the temporal aspect (i.e., the processing of upcoming inputs) but also the spatial dimension (i.e., the processing of upcoming inputs next-to-my-body). This is because accurate information processing of proximal sensory signals is key to the organism's survival in the here and now.

We narrowed our focus on tactile proximal interactions that are close to our bodies. We explored the special relationship between touch and body, and argued in line with the phenomenological accounts, that tactile experiences form a pervasive yet transparent experiential background, typically unnoticed and taken for granted. The key claim was that the body–world relationship remains tacitly and ubiquitously in the background and the sense of self is inseparable from this relationship.

To support this claim, we reviewed developmental empirical work that points to the idea that (discriminative and affective) tactile inputs play a fundamental role in constituting perceptual experiences *in utero* and in early life. We then explored the idea that our transparent experiential background includes also garments (i.e., objects and materials close-to-our-skin) that can be viewed as a “second skin” or “extended skin.” As such, they constitute what one may call, following Clark and Chalmers (1998) and Gallagher (2005) seminal formulations, an “extended” body-image and body-schema.

Our speculative proposal stipulates that proximal and tactile perceptual engagements with the physical and social world may form a pervasive yet transparent experiential bridge, typically unnoticed and taken for granted. Keeping this “bridge” open is essential in constituting the feeling of being real, present, and immersed in the world, especially when one's sense of self is shattered, as in the case of Depersonalization Disorder. If this is so, then future work and interventions need to focus on initiating and restoring dynamic and proximal engagements with our social and physical world in order to rebuild the “invisible” transparent yet essential basis of our sense of self and sense of presence in the world.

In line with this approach, we have recently piloted the sense of connection through touch in a set of experiential interventions with the microphenomenologist (Petitmengin et al., 2018). We have explored the aspect of quality of movements in textile experience further with designers through the Micro-phenomenology interview method^17^ (Petitmengin, 2006; Petitmengin et al., 2013, 2018) to go deeper into pre-verbal and non-verbal experiences (i.e., movement and touch behavior).

Briefly, participants explored with their hands and with closed eyes unknown objects for an extended time. They were then invited to share the experiences focusing on how they became aware of the interface of touch. Participants typically used images that were porous: “I felt fuzziness” often integrating social and spatial domains; “I felt open toward you.” Some would spontaneously describe a difference between touching and being touched. This distinction was subsequently frontloaded (Gallagher, 2003) into an exploration of another object. Participants were again invited to share these experiences, particularly noticing the distinction between touching and being touched, and on how sense of self and agency was experienced^18^. Such simple intervention appears to have the potentials to make visible those processes that connect us to our surroundings in ways that are both proximal and intimate. Future research will explore this in the context of depersonalization and other disturbances in embodied self-perception.

## Author Contributions

AC wrote the first draft of the paper. BP contributed with the textile/second skin section and provided comments on the first draft. AR and AF provided feedback on the final draft of the manuscript. All authors contributed to the article and approved the submitted version.

## Conflict of Interest

The authors declare that the research was conducted in the absence of any commercial or financial relationships that could be construed as a potential conflict of interest.

## Footnotes

^1^For example, Crane and French (2015) note that: “perceptual experience, in its character, involves the presentation (as) of ordinary mind-independent objects to a subject, and such objects are experienced as present or there such that the character of experience is immediately responsive to the character of its objects”.

^2^We recently had the chance to see this hypothesis at work during the ongoing COVID-19 pandemic: many of us experienced major difficulties to focus on abstract tasks, our attention being constantly attracted to the concrete conditions that threaten our lives and those of our loved ones.

^3^As Merleau-Ponty writes: “in so far as it sees or touches the world, my body can be neither seen nor touched. What prevents its ever being an object, ever being ‘completely constituted’ is that it is that by which there are objects. It is neither tangible nor visible in so far as it is that which sees and touches.” (Merleau-Ponty, 1962, p. 92).

^4^For example, some theorists argue that while we typically have access only to the mental representation's intentional content (something in the world which it is about) without noticing its non-intentional carrier properties (Moore, 1903; Harman, 1990; Tye, 1999), the process itself of constructing inner representations can become available to our introspective attention. Whenever we consciously direct our attention introspectively inwards, so to speak, the transparent processing of mental representations (typically taken for granted and hence “invisible”) becomes “opaque,” that is, “visible” and available to our attention (cf. the window metaphor described above).

^5^We do not claim that touch is the most fundamental proximal sensory channel, rather we outline its special role as a relational sense par excellence. We are grateful to one anonymous reviewer for pressing clarification on this point.

^6^Throughout this paper, we will use the terms “pregnant person” and “mother” interchangeably, irrespective of their self-identified gender.

^7^Interoception (the perception of bodily visceral signals) is taken to be uniquely related to the generation of subjective feelings, informing the organism regarding its levels of arousal and bodily needs (Craig, 2009; Seth, 2013). Even more importantly, and contrary to classic views of interoception as “the perception of the body from within,” the current notion of interoception is tightly linked to homeostasis. Within this framework, interoceptive signals are considered crucial in informing the organism regarding the homeostatic state of the body in relationship to experiences originating from both within the organism (e.g., cardiac and respiratory functions, digestion, hunger, and thirst) or outside it (e.g., taste, smell, affective touch, and pain).

^8^Substantial literature in the context of attachment formation also supports the facilitating role of touch in establishing the social bond between infant and caregivers (Ainsworth, 1979; Weiss et al., 2000).

^9^A gansey (or guernsey) is a type of knitted woollen jumper, historically known for being worn by fishermen around the coast of Britain. Their unique patterns represented a village and a family, so that in the case of a shipwreck or accident, the bodies could be identified and returned to their family for care or burial.

^10^This culture has reached the level of granting clothes and accessories even with world records, such as the most expensive ever pair of trainers, which was worn and signed by Michael Jordan, and sold at $560,000 at a Sotheby's auction held on May 17, 2020. For a variety of examples, see a recent feature. Available online at: <https://www.businessinsider.com/most-expensive-celebrity-memorabilia-items-sold-at-auction?r=US&IR=T#a-pair-of-nike-air-jordan-1s-game-worn-by-michael-jordan-in-1985-and-signed-by-the-player-sold-for-560000-in-a-sothebys-auction-1>.

^11^See de Vignemont and Iannetti (2015) for an interesting discussion on different forms of PPS.

^12^We are grateful to an anonymous reviewer for pressing clarification on this point.

^13^We are grateful to an anonymous reviewer for pressing extra clarification on this point.

^14^For example, the current social isolation provoked by the COVID-19 pandemic may reveal the importance of this tacit and “transparent” connectedness that we typically (before COVID) took it for granted.

^15^The other major classificatory system used in contemporary psychiatry is the ICD-10 (International Classification of Diseases, World Health Organisation). While there are some important differences between DSM and ICD, both largely agree upon the diagnostic criteria for DPD, which are the following: (a) persistent symptoms of DP/DR not occurring as part of another disorder or be directly substance-induced; (b) the individual should not be suffering from psychosis (which would imply a different diagnosis, such as schizophrenia). DSM adds the criterion (c) there should be significant distress and/or functional impairment. This seems appropriate, as otherwise it is hard to argue that the phenomena can usefully be seen as pathological (Medford et al., 2005).

^16^“Now I experience depersonalisation more of a lack of narrative; moments seem to melt away as soon as they have passed and life appears to pass as a series of unrelated frames. (…) You may be able to function, but creating a credible narrative for your life would be challenging” (Ciaunica and Charlton, 2018).

^17^This method combines psychology and phenomenology approaches to obtain a first-person, fine-grained verbal description of subjective experience that is generally inaccessible or difficult to articulate (Varela and Shear, 1999), and it reduces post-rationalization (Hogan et al., 2015). By using specific guidance (through questions and non-verbal cues), it helps people to become aware of nuances in the experience being described (Petitmengin et al., 2013). Hence, the method is presented as a “psychological microscope” (Bitbol and Petitmengin, 2013).

^18^A video detailing an excerpt of the experiment can be found here https://www.eer.info/activities/being-aware-sharpening-our-tools.
